# Animal welfare and society—Part 2, The viewpoints of a sociologist

**DOI:** 10.1093/af/vfac005

**Published:** 2022-03-17

**Authors:** Jocelyne Porcher

**Affiliations:** INRAE, UMR Innovation, 2 place Viala, 34060 , Montpellier, France

**Keywords:** animal husbandry, farm animals, industrialization, work

**Implications**

• Animal husbandry and animal industrial production refer to two different worlds in which work has neither the same meaning nor the same purpose.

• Animal welfare issues are powerless to change the lives of farm animals. This leads to the radicalization of anti-farming positions.

• The question of work is conducive to thinking about, understanding and transforming our relationships with farm animals and, more broadly, with domestic animals.

• Working with animals is not just about producing, it is also about living together.

• Farm animals need respect more than “welfare.”

## What is Animal Welfare (Definition)?

In spite of the anteriority of the concept and the abundant research to which it gave rise, I do not consider that “animal welfare” is an operational concept, nor even a simple concept, i.e., scientifically consensual, robust, useful, and necessary. I further think there is an urgent need to move from the issue of “animal welfare” to that of “animal work.” This is for two main reasons. The first is that well-being is a state that is subjective and transitory, that is, succeeding another state, like sun after rain, unless one is constantly on Prozac (drug used to cure depressive states). To be well every hour of the day and every day of the year is impossible. Life is both pleasure and suffering. The second is that the welfare of the animals in question here, domestic animals (cows, goats, chickens, dogs, horses, etc.), is contextualized through work. Welfare strongly depends on the living conditions of the working animals, including at work. Our demands as human beings in relation to work are not “welfare,” for that, it is better to choose a type of activity more directly favorable for this, or to go to the hammam, for those who like hammam; rather, it is the possibility to do a job that has meaning, to be able to do it well and to be recognized for it.

There is no concept of work. This is why the psychodynamics of work—the discipline on which my research is based—proposes to focus on the *working*, i.e., the subjective investment of individuals in the activity, the “someone” who works. This makes it possible to bring to light the fact that working means producing, but it is not only producing, it is also producing oneself and living together. What I call “rationalities” of work. *Working* is to invest one’s intelligence, subjectivity, and affectivity in a productive activity. It is much more the question of the meaning of the activity that counts than that of “welfare.”

It is the same for domestic animals. What meaning can the animal give to its existence? Taking an interest in the work of animals makes it possible to question working time, breaks, off-duty time, and also retirement. The question is therefore not that of “animal welfare,” or of “happy management,” but: what are good working and living conditions for working animals? Animals are our collaborators at work. We produce goods and/or services together. However, cows are cows, not human beings. As von Uexküll (1984) pointed out, there is a human world and there are animal worlds. Yet, in the case of domestic animals, their world is also partly human. Their lived world is located at the interface of their own world and our human world. The stake of the work lies in this interface. Good working conditions are those that make sense in the human world and are consistent with the world of the cow, such as milking and grazing. Milking is a strong moment of encounter with humans for the cow. It is her relationship with humans that gives meaning to the milking situation. The cow does not *produce* milk at that very moment, but in the milking parlor, she knows and respects the rules of the work. She knows the rhythm of the machine, the specific noises, the tension normally produced on her udder, the farmer’s voice, and what he/she is saying—maybe even the radio station he/she listens to. When on pasture, she is in her own world, with her conspecifics. She knows the grass, the smell of meadows, and trees. She walks at her own pace, if she wants, with whomever she wants. Good working and living conditions at work are central to a successful link between the animal world and the human world. However, it is not “welfare” that is the key of the relationship, it is respect. Cows do not ask for “welfare.” They demand respect, from birth to death (Porcher, 2017). Respecting animals means taking them into consideration, acknowledging their presence, their existence, their intelligence, and their skills, and so thinking about the rules of work in terms of all this.

It should be remembered that the problem of “animal welfare” appeared in Great Britain in the 1960s with the book by Ruth Harrison, “Animal machines” in response to the development of industrial systems. In the same way, animal protection associations emerged in the 19th century with industrial capitalism, even if this economic system has not had a monopoly on industrial violence against animals: the question of “animal welfare,” as addressed by the scientific issue of the same name, was not asked to animal husbandry but to its industrial avatar. It is in the industrial world, and because it considers animals as things, that violence against animals has been trivialized by the organization of work. Violence against animals has always existed because animals are part of social relationships and these are historically conflictual, but we have moved from individual violence to systemic violence linked to the industrial organization of work. Cows could be victims of individual violence. Today, millions of cows are victims of organizational violence.

The persistent question of “animal welfare” in the industrial and capitalist field may increase the risk that the work with animals increasingly tends towards dead work rather than living work. Here are two examples. Consider that for reasons of “biosecurity” and “animal welfare,” industrial systems are preferable to free-range systems. Locked up, animals are supposedly less likely to be affected by viruses carried by wild animals (because nature is the enemy of animals), therefore less sick and therefore in better “welfare” condition. The animal as an industrial thing, theorized as part of dead labor, a machine, is therefore preferable to considering the animal involved in living work conducted by animals and by humans capable of resisting attacks.

The other risk, collectively much greater, is to switch completely to the side of dead labor by doing without animals, for example, in the production of “cultured meat.” In this case, the “animal welfare” would be supposedly optimum. As Mark Post, a pioneer in *in vitro* meat research, says, in his lab, the cow will be “happy forever.” Saying this, if we bypass the issue of work, is also evacuating the issue of happiness at work. Because for Mark Post, the happiness of the cow is limited to the fact of not being killed (in theory only, because it is very likely that she will end up euthanized). It does not take into account the work that the cow will have to do and the satisfaction that this may bring her. In fact, most of what matters to a cow will be absent from the laboratory: herd life, sexuality and the birth of a calf, grazing, finalized and meaningful relationships with fellow creatures and with humans. She will be a “donor” of cells. Laboratory animal work not intended as such but meeting ad hoc “animal welfare” standards.

## Does Animal Welfare Matter? If Yes, Why? If No, Why Not?

Considering I do not find the concept of animal welfare relevant, I will answer by replacing it with the word respect, that is to say the consideration, the recognition of the existence of animals. Is respect for domestic animals important? Yes. Very important. What does that mean, respecting animals? Regarding farm animals, respecting animals means going back over 150 yr of industrialization of animal husbandry. When in the middle of the 19th century, agronomists, veterinarians, and notables invented zootechnics, the science of the exploitation of animal machines, they began a conceptual process of reification and specialization of animals which would lead a century later to the development of systematized animal production, that is, industrial systems as a hegemonic model. In clear terms, this means treating animals as industrial objects and to produce animal material—bovine and porcine material, amongst others—from animals. The industrial organization of work in animal production makes that animals are integrated in the register of dead work, that of machines and things. It is for this reason that male chicks can be crushed, underproductive piglets gassed, “worthless” dairy calves destroyed. They are nothing. They do not count more than a bolt in the system. This reified status of animals is consubstantial with animal productions. These are not about raising animals to live with and feed people. They have only one rationality and it is economical. Animal productions aim to make a profit. They have no other purpose.

In this industrial world, respecting animals is therefore impossible. As soon as you integrate animals into industrial production processes, you take away their character as living beings. In the industrial organization of work, animals are machines for production (sows, cows, laying hens) or objects produced (pigs for meat, poultry for meat). It is not possible to do “factory farming” (Porcher, 2011). This term is an oxymoron. Animal husbandry is incompatible with industry, because animal husbandry does not only have an economical rationality, it also has relational, moral, and aesthetic rationalities. In animal husbandry, the objective is to raise the animals. To have them born, have them grow up, and to let them die without ceasing to reserve to them our relational and moral commitments. This is why raising animals also has the effect of uplifting us humans. By raising animals, individually and collectively, we are raising ourselves. We rise in skills, in affectivity, in subjectivity, and in humanity.

While respect is not an issue in animal production, given its intrinsic violence, it is in animal husbandry. Indeed, in the globalized hegemonic context of animal production, raising animals is a struggle, especially against industry and against the administration. As an American farmer put it, “Everything I want to do is illegal” (Salatin, 2013). You could add, anything I want to do *right* is illegal. For example, choosing the breed of animals I want to raise (because, for example, slaughterhouses do not accept heavy, non-compliant animals with equipment designed for standard animals), choosing their diet or the way you treat them because the natural products you use are prohibited, choosing the living conditions at work that I want to give them, choosing to slaughter them on the farm, choosing to process and sell my products as I see fit. In other words, working with respect for animals, for oneself and for our fellow citizens, that is the goal of animal husbandry and that is what is made difficult by contemporary economic and political conditions.

Respect for animals is also important because it involves using work skills normally crushed by animal productions, especially the intelligence of animals. In animal husbandry, animal farmers rely on the intelligence of animals to work and they entrust them with the part of the work that is due to them. For example, animal farmers will not cut the grass to provide it to zero grazing cows. They let graze cows, goats, or sheep and let them choose for themselves what suits them, including plants or brambles that they consume for reasons only known to them. Animal husbandry is a relationship of donations. It is not of the absolute Christian gift but of the Maussian gift (Mauss, 1999). The anthropologist Marcel Mauss brought to light the place of the gift in the construction of the social bond. It is about giving, receiving, and rendering. The gift combines interest and disinterestedness, obligation, and freedom. This entry through donation makes it possible to understand not only the intersubjective relations between farmers and animals—and the fact that animal farmers devote their entire lives to animals—but also the collective relations of domestication. What has linked us to animals for ten millennia is gift and debt. The donation cycle was broken by the industrialization of animal husbandry. Hence, it is not about “setting free” animals and getting rid of our debt to them, but about resuming the cycle of giving. To live up to the animals, when industrialization has brought us down.

## Is It Acceptable to Eat Animals? If Yes, Why? If No, Why Not?

We are animals and, like others, we are omnivorous, which also means carnivorous. However, unlike predators like felines or scavengers like jackals or vultures, we do not eat whole animals (except oysters, for example) or corpses. Among others, we eat products from animals that we have killed (beef) or that we may be killing (laying hens, dairy animals). We eat meat, but we do not only eat meat. We consume milk and eggs. Consuming milk or eggs indirectly contributes to the breeding and slaughter of animals. This is why vegetarianism, as part of a compassionate out-of-farm thinking system, is a moral aporia that logically leads to veganism. Within the framework of a complex thinking system centered on the relation to animals, it leads on the contrary to a commitment for a breeding respectful of animals, humans, and nature.

So is it morally, practically acceptable to consume animal products? My answer is yes. The answer has been yes for ten thousand years in human societies, whether they are hunter-gatherer societies or farmers. Why? I will not respond here generally in support of the work of anthropologists of domestication or sociologists of food but from real work experience, that is to say my own animal farmer experience and the experience of hundreds of farmers that I have met during my 25 yr of research. Before being a farmer, like everyone else, I bought my food in stores, meat, milk, eggs, fruits, vegetables, cereals, etc. I knew that farmers produced all this but I did not know what it meant to them. It was by making a garden myself, growing vegetables, then having animals, because I wanted to live with animals, dogs, chickens, and sheep that I understood the meaning of it all: the link between hens and eggs, chickens and poultry, geese and goose down in the quilt, lambs, and cheese. Things, life and death, pleasure and suffering, attachment and detachment are not separate. On the contrary, everything is linked and it is the links between all these activities that make sense. If you separate them, the meaning disappears and eating animal products seems an unnecessary cruelty. If one day we will learn more about the sensitivities of plants (Mancuso and Viola, 2020), and if we do not relate things to each other, inevitably, pulling carrots will seem as brutal to us as chopping down a tree. Then, unless we base our food resources on food biotechnology and cellular agriculture, we will have to resolve to starve in a very ethical way (Porcher, 2020).

Why is eating animal products including meat not a crime and is not cruel? Here I rule out the violence of production and industrial slaughter. Industrial systems are not acceptable and consuming the products that come out of them is not either. I also leave out the context of countries where people are suffering from famine and where asking whether it is right or wrong to eat animal products would be particularly indecent, if not cynical. I am talking here about animal products, to put it clearly, farm animal husbandry products, resulting from work relationships with animals that have been respectful, as much as can, given the increasing constraints that the state and industry place on breeders.

When we raise animals, for example, chickens, geese, and sheep, as I have done myself, we are in daily proximity to animals. We allow them to be born, we treat them if they are sick, we make sure that they are well nourished, that they live in good physical and mental conditions, that they are happy. Twenty-four hours a day, we are at the service of the animals, because we are with them in a relationship of giving, and we must live up to what they give us, for some their lives. An animal that we have raised, to which we have given the best possible life, we know we will kill it, skin it, cut up its carcass, eat the meat, share it, or sell it. We will tan his skin and make it into a leather garment or a satchel. We are going to carve its horns into an object of art. And, yes, that is right and moral. Of a morality that is not transcendent and supposedly universal (we must not kill our neighbor), but which is linked to work and to our real life.

Animals are not our neighbors (to claim that would be to deny their uniqueness and to show contempt for them) and we are not theirs. We are not similar to them and they do not expect us to be. To live and work together, we do not need them to be neighbors, but companions, who are well in their own world, well in our common world. In this work relationship, not compassion, but respect is foundational. However, we have to eat to live and animal products are rich and precious. Therefore, we must not produce or eat more than necessary and remember when eating yogurt or meat that this food is not nothing, that it represents human and animal work, and even more, even the life of an animal.

Remembering the animal behind the meat is not a repulsive observation but on the contrary a recognition that must be reiterated. What is important are the conditions of life and death of animals. For a majority of farm animals, leaving work leads to the slaughterhouse. For others, it leads to a retreat from the herd or to a new work situation. Retirement is in fact an exit from work. The living conditions of the animals must then be considered from the point of view of maintaining health and avoiding suffering due to aging. With regard to calves, lambs, and kids whose birth is related to milk production, there are two issues. The first is the extension of their life expectancy, and the second is, because it is impossible to keep all these animals, the conditions of their slaughter. Life expectancy depends on the possibility that farmers may have to create micro-sectors independent of animal production and major distribution channels. It also depends on the ability of consumers to agree to change their consumption habits.

Regarding the slaughter of animals, the challenge is to avoid industrial slaughterhouses and to develop slaughter systems managed by the farmers themselves, that is, not intended to be profitable but to be useful. It is about giving back autonomy to farmers and adapting slaughter modalities to the breeding system, for example, through slaughter on the farm. The slaughter of animals is a necessity in animal farming related to the major fact that breeding systems are finite systems and that if animals are born, animals must leave. This is also the case with our earth system and the worst thing that could happen to us would be to become immortal. However, the death of animals is not a pleasure for any farmers, especially those animals that have lived a long time on the farm. Many farmers constitute a sub-herd of retirees, who no longer work but who follow the herd. Sometimes they give their animals to a sanctuary, for example, ewes or hens out of work may leave to be kept by a private individual. This, despite the dominant productivist ideology and the economical interests of farmers. As Jankélévitch (1977) writes, you have to be alive to die. It is life that matters, life lived, its intensity and beauty is more important than its duration. This was also emphasized by de Montaigne (2002): what is important is what we do with our life. We must give the possibility to animals to fulfill theirs, with us.

## Do All Animals Deserve Equal Consideration in Terms of Animal Welfare?

We are talking about domestic animals here. While “wild” animals are also entitled to receive our consideration, their welfare does not depend on our working relationships. We do not work together even though we live on the same planet devastated by an economic system, which affects them dramatically. Are there any domestic animals, that is, animals with whom we are in a working relationship, that are entitled to more consideration and respect than others, and why? Should we have more respect for dogs than for chickens, for hens than for carp, for carp than for silkworms? Why? Because, as the “great apes” project underpins, would they have greater proximity to humans, greater cognitive abilities, greater sensitivity? In my opinion, no. There is no reason. All animals are entitled to respect. That is, all must be taken into consideration in their existence. A chimpanzee has no more right to consideration than a pig, no less. But we understand, however, that this respect is not viewed the same way by all animals. Respecting a cow and respecting a silkworm do not have the same consequences for animals, for ourselves, and for our relationship. Humans are mammals, and we have much greater empathy and possibilities for communication with other mammals. As the majority of animal farmers claim, we talk to the cows and they understand us. They express themselves and we understand them. We live together in the interface of worlds that have much in common. This is not the case with silkworms.

What about the so-called “companion animals” like dogs or farm animals like pigs? In fact, regulations on “animal welfare” mainly concern farm animals given their dismal conditions in industrial systems. “Companion” animals are mostly protected against individual mistreatment. On both sides, in fact, what animals can face is poor working conditions, given that both the dog and the cow are at work. One supplies a service, often the company, the other food. Both have a right to a decent and meaningful work life. However, this is not what cows or pigs in industrial systems have. It is not what many dogs who live in apartments have.

The problem is that for both sides, the issue of work is not taken into account. However, to consider all animals fairly means first, to take into account their respective working conditions. A cow works (Porcher and Schmitt, 2012). A laying hen is working. A horse works. A companion dog works. With what organization? What rules? What consequences? The same questions arise for all domestic species although the concrete answers may differ a little (Porcher and Estebanez, 2019). However, for all of them, the following questions arise: what relationship to nature? What learning? What production performance is required? What relations with humans? What life expectancy? What end of life? By asking this question, we perceive that it is less the animal species that makes the difference than the production system. There is as much similarity between a laying hen raised in the open air in a small group and a town horse that leads the children to school and finds its fellows in the paddock in the evening; as between a cow in zero pasture locked up 24 h a day and a dog stuck in an apartment from morning to evening. The dog is not better off because he is a dog. While he too is entitled to respect and to have his work considered, the effort he makes to be a “companion” dog, because keeping company is work, it is hardly natural. All animals are entitled to our consideration and to be recognized for the place they occupy in our lives, dog, pig, fish, or horse. We owe them all respect and concern.

## About the Author

Jocelyne Porcher is a zootechnician and sociologist, research director at INRAE (PhD in zootechnie, HDR in sociology). Before becoming a researcher, she was an animal farmer, an employee in industrial pig production, and a technician in organic farming. Her research focuses on the working relationship between humans and animals. She is the author of a dozen books on the subject. She is particularly interested in the affective relations between humans and animals in the workplace, in suffering and pleasure at work, in the slaughter of animals and alternatives to industrial slaughter, in animal labor, and in cellular agriculture. She also produces a critical sociology of the “animal cause” and its logic of development within the capitalist system.
